# An Atypical Case Presentation of Babesiosis

**DOI:** 10.7759/cureus.60036

**Published:** 2024-05-10

**Authors:** Dannielle Allen, Leila Getto

**Affiliations:** 1 Emergency Medicine, ChristianaCare, Newark, USA

**Keywords:** thrombocytopenia, hemolytic anemia, differential diagnosis, babesiosis, tick-borne illness

## Abstract

Babesiosis is a tick-borne illness that can cause a wide variety of symptoms based on the severity of the disease. Mild presentations can be difficult to identify, and as a result, treatment may be delayed. A 75-year-old male presented to the Emergency Department (ED) with weakness, leg pain, and a fall. He was found to be febrile and tachycardic on arrival, and lab work revealed thrombocytopenia and acute renal dysfunction. He was admitted and found to have a Babesiosis infection, requiring treatment with red blood cell exchange and plasma exchange therapy. Tick-borne illnesses should be included in the differential even in low-risk populations and non-endemic regions due to the severity of disease complications.

## Introduction

Babesiosis is a tick-borne illness caused by intraerythrocytic protozoal parasites that can result in a wide variety of symptoms based on the severity of the disease. Of the many species of Babesia, only a few are known to cause human infections, with *Babesia microti* being the predominant species [1,2]. These infections are usually seen in the Northeastern and upper Midwestern regions of the United States as well as in Southwestern China [1-3]. Less common Babesia species are endemic in Northeastern China and Europe, and sporadic cases have been reported worldwide [2-4]. Babesiosis is most commonly transmitted to humans through tick vectors, *Ixodes scapularis* or the deer tick being the most common [1,2]. As the parasite replicates intracellularly within erythrocytes, it will lyse the erythrocytes and lead to hemolytic anemia and, depending on the severity, tissue hypoxia [5].

The incubation period usually ranges from one week to four weeks but has been noted to be longer [2,6,7]. While infected people usually present with symptoms, studies have shown that up to one-third of infections may be asymptomatic [8]. Asymptomatic infections are important to note, as blood donors could potentially transmit the infection [4]. As of May 2019, the FDA issued recommendations for all donated blood in endemic areas to be screened for babesia infection [4]. Mild presentations can be difficult to identify, and treatment may be delayed due to this. More severe infections have been seen in newborns, older adults, and immunocompromised individuals [2,4]. Severe infections may lead to disseminated intravascular coagulation (DIC), liver and renal failure, respiratory failure, and congestive heart failure [5]. In the hospitalized population with severe infection, mortality was as high as 10% [4].

## Case presentation

A 75-year-old male with a past medical history of hypertension and hyperlipidemia presented to the Emergency Department (ED) in the summer season for evaluation of weakness and fall from standing while walking in his home. He also reported intermittent stabbing pain in his lateral left thigh, mild swelling of the left leg, and generalized weakness for one week. He reported feeling “off” over the past few days and had been treating his symptoms with acetaminophen at home without relief. He denied presyncopal symptoms, head strikes, loss of consciousness, or injuries from the fall. He denied any known fevers at home, chills, shortness of breath, chest pain, neck pain, back pain, incontinence of bowel or bladder, prior injuries, or recent travel. The patient had seen his primary care provider a few days prior for these symptoms and was sent for outpatient lab work as well as a duplex ultrasound incorporating grayscale and color Doppler imaging of his left lower extremity. His ultrasound was negative for acute deep vein thrombosis, and lab work was only remarkable for a hemoglobin of 12.3 g/dl and platelet count of 116 x10E^3^/uL.

On arrival to the ED, the patient’s initial vital signs showed he was febrile at 39.2°C, tachycardic to 116 beats per minute, had a respiratory rate of 22 breaths per minute, oxygen saturation of 97% on room air, and blood pressure of 162/122 mmHg. Physical examination noted a non-ill-appearing male who appeared to be his stated age. He had a regular rate and rhythm on cardiac exam and clear breath sounds bilaterally. He had tenderness to palpation of his lateral mid-left thigh with 1+ edema of the left lower extremity without evidence of surrounding erythema, petechiae, purpura, abscess, or wounds to the area. The patient was alert and oriented to person, place, and time and without focal neurological deficit, although the patient did appear to be slow to answer questions. The patient did experience a few episodes of oxygen desaturation to 88% on room air during his stay in the emergency department and was placed on 2 liters nasal cannula.

The patient's initial laboratory testing revealed mild anemia, thrombocytopenia, and renal dysfunction (Table 1). Based on the patient's symptoms and initial lab testing, the differential diagnosis included myositis, thrombotic thrombocytopenic purpura (TTP), DIC, necrotizing fasciitis, pulmonary embolism (PE), viral illness such as COVID-19 or influenza, pneumonia, and urinary tract infection. Given that the differential diagnosis included TTP and DIC, additional coagulation laboratory studies, including lactate dehydrogenase (LD) and DIC panel were ordered.

**Table 1:** Initial laboratory results WBC: White blood cell; HGB: Hemoglobin; HCT: Hematocrit, RBC: Red blood cell, MCV: Mean corpuscular volume; MCH: Mean corpuscular hemoglobin; MCHC: Mean corpuscular hemoglobin concentration; RDW: Red blood cell distribution width; RBC Nuc: Nucleated red blood cell; Manual ANC: Manual neutrophil count; Lymph: lymphocyte count; Estimated GFR: Estimated glomerular filtration rate; INR: International normalized ratio; PTT: Partial thromboplastin time; LD: Lactate dehydrogenase.

Lab Test	Value (reference range)	Lab Test	Value (reference range)
WBC	6.6 x 10E^3^/uL (3.9-10.6)	Glucose	131 mg/dl (70-99)
HGB	11.0 g/dl (13.3-17.7)	Lactate	1.8 mmol/L (0.5-2.2)
HCT	31.6% (40-52)	Sodium	129 mmol/L (136-146)
RBC	4.20 x 10E^6^/uL (4.4-5.9)	Potassium	4.9 mmol/L (3.5-5)
MCV	75.2 fl (80-100)	Chloride	98 mmol/L (98-107)
MCH	26.2 pg (26.5-34)	Total carbon dioxide	19 mmol/L (24-32)
MCHC	34.8 g/dl (31.5-36.3)	Blood urea nitrogen	32 mg/dl (8-22)
RDW	16.4% (11.5-14.5)	Creatinine	1.69 mg/dl (0.7-1.3)
Platelet	52 x 10E^3^/uL (150-400)	Estimated GFR	42 ml/min/1.73 sqm
RBC Nuc	<1 per 100 WBC	Anion Gap	12 (4-12)
Manual ANC	4.7 x 10E^3^/uL (1.8-6.6)	Creatine kinase	761 unit/L (40-250)
Absolute Lymph Manual	1.2 x 10E^3^/uL (0.9-4.4)		
Absolute Atyp Lymph	0.1 x 10E^3^/uL (<0.1)	Protime	13.8 seconds (10.3-12.9)
Absolute Plasma Cells	0.2 x 10E^3^/uL (<0.1)	INR	1.2 (0.9-1.1)
Absolute Mono Manual	0.4 x 10E^3^/uL (0.1-1.1)	PTT	35 seconds (22-36)
Manual Neutrophils	61.3% (50-60)	Thrombin time	22 seconds (16-25)
Manual Bands	10.1% (0-5)	Fibrinogen	670 mg/dl (180-410)
Manual Lymph	17.6% (25-40)	D-dimer	2979 ng/ml (<244)
Manual Atyp Lymph	1.7% (<0.1)		
Manual Plasma Cell	3.4% (<0.1)	Covid-19	Negative
Manual Mono	5.9% (4-10)		
Anisocytosis	Slight	Haptoglobin	<3 mg/dl (32-197)
Hypochromasia	Slight	LD	737 unit/L (107-270)
Polychromasia	Slight		
Target Cells	6-15%		

Chest radiographs, as well as radiographs of the left hip, femur, and knee were negative for any acute abnormalities. A computed tomography scan (CT) of the left lower extremity and computed tomography scan with angiography (CTA) chest pulmonary embolism protocol were negative. The patient was treated with acetaminophen and empiric intravenous ceftriaxone 1 g and vancomycin 1250 mg. The patient was admitted to the hospital for further work-up and management of a fever of unknown origin.

On the first day of hospitalization, blood parasites were noted to be present on the patient’s complete blood count (CBC). Given this finding, the team initiated testing for tick-borne infections including Lyme disease, Ehrlichiosis, Leptospirosis and Babesiosis. Infectious disease was consulted, and the patient was started on a 10-day course of azithromycin and atovaquone for the working diagnosis of Babesiosis. 

The patient’s renal function, anemia, thrombocytopenia and mental status progressively worsened and by hospital day 3 the patient was transferred to the Intensive Care Unit. Hematology was consulted and the decision was made to treat with a 10-unit red blood cell exchange and plasma exchange therapy. The following day, the patient was found to have improving kidney function and his anemia and thrombocytopenia stabilized. The percentage of parasitemia had decreased to 1% from a maximum of 22% on Day 1 of admission. Polymerase chain reaction (PCR) testing for Babesiosis resulted in a positive result for *Babesia microti*. The patient showed progressive improvement in his central nervous system, renal, and hematologic function and was discharged home after another three days of hospitalization.

## Discussion

Babesiosis is a tick-borne illness that can present in a variety of ways. There are many case studies describing unique presentations of tick-borne illnesses, although these cases usually occur in individuals at extremes of age or in immunocompromised individuals [5,6,8]. Our patient was not immunocompromised and had limited risk factors for tick-borne illnesses. The majority of Babesiosis cases on the East Coast of the United States have been found in Rhode Island, Connecticut, Massachusetts, Maine, New York, and New Jersey [9]. There were only four reported cases of Babesiosis in Delaware residents in the year 2020 [9].

In the hospital it was also determined that the patient had a dog, which was suspected to be the carrier of the tick, but this theory was not confirmed. We had not asked about additional risk factors that may have led to a tick-bite, but in hindsight these questions are vital to incorporating into practice. 

Patients who present to the emergency department with vague symptomatology, frequent admissions, or for whom a diagnosis has been difficult to determine may require more broad workups. Babesiosis infection can present similarly to a flu-like illness or non-specific viral illness [5]. Initiating a workup for patients in which you have a concern for tick-borne illnesses is reasonable for patients in the emergency department [5]. These panels will take days to result, and if the patient is well enough to be discharged home, their care can be expedited through their primary care provider if testing has already been sent.

When initiating treatment in the emergency department for tick-borne illness, it is important to remember that Babesiosis is not treated with doxycycline, unlike Lyme disease, Ehrlichiosis, Anaplasmosis, and Rocky Mountain spotted fever [4,10]. The treatment for Babesiosis is based on symptom severity [4,10]. Patients with a mild infection classified as parasitemia <4% will be treated with azithromycin 500 mg once followed by 250 mg daily plus atovaquone 750 mg twice daily for 7-10 days [4,10]. For patients that have a severe infection with parasitemia >4%, treatment recommendations include intravenous azithromycin daily plus atovaquone 750 mg twice daily [4,10]. Additionally, clindamycin 600 mg and quinine 650 mg orally three times daily for 7-10 days can be used if there are allergies [4,10]. For patients with severe disease, as in our patient, an exchange transfusion is indicated if there is >10% parasitemia, hemoglobin <10 g/dL, or evidence of pulmonary or hepatorenal impairment [4,10].

## Conclusions

As Emergency Medicine physicians, we are tasked with synthesizing a patient’s history, creating differential diagnoses, initiating a workup, and determining the safest disposition despite not always having the definitive diagnosis. When patients present with vague symptoms, it is important to keep a broad differential. In this case, it could have been beneficial to inquire if the patient spent time outdoors or had any pets or other means by which he may have been exposed to a tick. Tick-borne illnesses should not be left off of the differential, even in non-endemic regions, and especially during the summer months, as they pose a potential for multisystem organ failure and even death without the proper treatment. We may not have the means to diagnose tick-borne illnesses in the emergency department, but we can have the thought process to risk stratify patients who need further workup. Early diagnosis is important in severe cases where multidisciplinary treatment strategies may be required. 

## References

[1] Vannier E, Gewurz BE, Krause PJ (2008). Human babesiosis. Infect Dis Clin North Am.

[2] Waked R, Krause PJ (2022). Human babesiosis. Infect Dis Clin North Am.

[3] Kumar A, O'Bryan J, Krause PJ (2021). The Global emergence of human babesiosis. Pathogens.

[4] Lobo CA, Singh M, Rodriguez M (2020). Human babesiosis: recent advances and future challenges. Curr Opin Hematol.

[5] Varshavsky T, Cuthbert D, Riggs R (2021). Babesiosis in the emergency department: a case report. J Emerg Med.

[6] Patel JK, Tirumalasetty K, Zeidan B Jr, Desai P, Frunzi J (2020). A case report of babesiosis seen outside of its endemic area and incubation period. Cureus.

[7] Krause PJ, Auwaerter PG, Bannuru RR (2021). Clinical practice guidelines by the Infectious Diseases Society of America (IDSA): 2020 guideline on diagnosis and management of babesiosis. Clin Infect Dis.

[8] Hajicharalambous C, Rattu M, Leuchten S (2018). A walk in the park: a case of babesiosis in the South Bronx. Clin Pract Cases Emerg Med.

[9] (2024). Surveillance for Babesiosis- United States, 2020, Annual Summary. https://www.cdc.gov/parasites/babesiosis/resources/Surveillance_Babesiosis_US_2020d.pdf.

[10] (2024). Parasites-Babesiosis. https://www.cdc.gov/parasites/babesiosis/health_professionals/index.html#tx.

